# Different impact of bovine complement regulatory protein 46 (CD46_bov_) as a cellular receptor for members of the species *Pestivirus H* and *Pestivirus G*

**DOI:** 10.1080/22221751.2021.2011620

**Published:** 2022-01-04

**Authors:** Elena Leveringhaus, Gökce Nur Cagatay, Juliane Hardt, Paul Becher, Alexander Postel

**Affiliations:** a Institute of Virology, University of Veterinary Medicine Hannover, Hannover, Germany; b Coriolis Pharma Research GmbH, Martinsried, Germany; c Department of Biometry, Epidemiology and Information Processing, WHO Collaborating Centre for Research and Training for Health at the Human-Animal-Environment Interface, University of Veterinary Medicine Hannover, Hannover, Germany

**Keywords:** Bovine viral diarrhoea virus, HoBi-like pestivirus, giraffe pestivirus, CD46, cellular receptor, viral entry, heparan sulfate, cell culture adaptation

## Abstract

The genus *Pestivirus* within the family *Flaviviridae* comprises highly relevant animal pathogens such as bovine viral diarrhoea virus 1 and 2 (BVDV-1 and -2) classified into the two species *Pestivirus A* and *Pestivirus B*, respectively. First described in 2004, HoBi-like pestiviruses (HoBiPeV) represent emerging bovine pathogens that belong to a separate species (*Pestivirus H*), but share many similarities with BVDV-1 and -2. Additionally, two giraffe pestivirus (GPeV) strains both originating from Kenya represent another distinct species (*Pestivirus G*), whose members replicate very efficiently in bovine cells. In this study, we investigated the role of bovine complement regulatory protein 46 (CD46_bov_), the receptor of BVDV-1 and -2, in the entry of HoBiPeV and GPeV. For this purpose, bovine CD46-knockout and CD46-rescue cell lines were generated by CRISPR/Cas9 technology and subsequent trans-complementation, respectively. Our results provide strong evidence that the impact of CD46_bov_ differs between viruses belonging to *Pestivirus H* and viruses representing *Pestivirus G*: CD46_bov_ revealed to be a major cellular entry factor for HoBiPeV strain HaVi-20. In contrast, GPeV strain PG-2 presented as largely independent of CD46_bov_, suggesting a different entry mechanism involving other molecular determinants which remain to be identified. In addition, we demonstrated that, similar to BVDV-1 and -2, virus isolates of both *Pestivirus H* and *Pestivirus G* are able to adapt to cell culture conditions by using heparan sulfate to enter the host cell. In conclusion, our findings show that different bovine pestiviruses use diverse mechanisms of host cell entry.

## Introduction

The genus *Pestivirus* within the family *Flaviviridae* comprises important animal pathogens such as bovine viral diarrhoea virus 1 and 2 (BVDV-1 and -2) and classical swine fever virus (CSFV), the causative agents of the notifiable animal diseases bovine viral diarrhoea (BVD) and classical swine fever (CSF), respectively [1,2]. Pestiviruses possess a positive-sense single-stranded RNA genome that encodes for the core protein (C), the three envelope glycoproteins E^rns^, E1 and E2, and eight non-structural proteins [3].

Over the last 20 years, a growing number of new pestiviruses have been discovered, leading to a revised taxonomy: the well-established pestiviruses BVDV-1, BVDV-2, CSFV and Border disease virus (BDV) are now assigned to the species *Pestivirus A*, *Pestivirus B*, *Pestivirus C* and *Pestivirus D*, respectively [4–6]. The existence of another pestivirus species comprising bovine viruses was first suggested in 2004 when an atypical pestivirus designated D32/00_“HoBi” (HoBi) was isolated from a batch of fetal calf serum (FCS) from Brazil [7]. Subsequently, HoBi-like pestiviruses (HoBiPeV), later acknowledged as members of a new species termed *Pestivirus H* [4], were detected in commercially available FCS batches from Canada, the USA, Mexico and Australia [8] as well as in healthy and diseased cattle in several countries [9–15]. Clinical symptoms are similar to BVD caused by BVDV and include respiratory disease [16,17] and mucosal disease (MD)-like syndrome [18,19]. Infection during gestation can lead to reproductive disorders, e.g. abortion and birth of persistently infected (PI) calves [20,21]. According to a case report from Italy, outbreaks caused by HoBiPeV can lead to substantial economic losses [22]. Furthermore, there is a considerable risk of contamination of biological products containing FCS, e.g. vaccines, which could lead to further distribution of HoBiPeV [23,24]. Thus, viruses belonging to *Pestivirus H* must be considered highly relevant emerging livestock pathogens that pose a threat to animal health as well as biosafety of biological products.

Another pestivirus readily replicating in bovine cells was named giraffe pestivirus (GPeV) after being isolated from a diseased giraffe in Kenya in the 1960s (Giraffe-1 / H138) [25]. As the giraffe showed MD-like symptoms, it can be speculated that GPeV has similar pathogenic properties as BVDV-1, BVDV-2 and HoBiPeV. It is the first member of the species *Pestivirus G* [4]. The second and only other member of this species known to date, designated PG-2, was discovered in the 1990s in Switzerland as a contaminant of a bovine cell culture many years after it was prepared from a local cattle and local FCS in Kenya, demonstrating the ease by which such virus strains were probably propagated unknowingly worldwide [26]. Their close genetic relationship with a nucleotide identity of 82% and the ability of both strains to efficiently replicate in bovine cells provide evidence that cattle are a susceptible host of *Pestivirus G* [27,28]. It seems likely that GPeV are not giraffe-specific pathogens, but ruminant pestiviruses that were circulating unnoticed in Kenyan cattle at the time. It cannot be excluded that further GPeV strains or other related viruses are still circulating in livestock and/or wild ruminants.

Pestiviruses enter their host cells via receptor-mediated endocytosis [29–31]. For BVDV-1 and -2, bovine complement regulatory protein 46 (CD46_bov_) has been identified as a receptor [32]. CD46 is a ubiquitously expressed “multitasker” as it is a regulator of both the complement system and adaptive immunity [33]. It consists of several domains: four complement control proteins (CCP1-CCP4), a highly variable Serine/Threonine/Proline-rich region (STP), a transmembrane domain (TM) and a cytoplasmic tail (CT). Viral entry of BVDV-1 is mediated by the interaction of E2 with the CCP1 domain [34]. Moreover, a study has shown that CD46_bov_ variants with long CTs shift cell permissivity to infection with BVDV-1 strain NADL [35]. Still, there are various indications of the existence of at least one alternative receptor for BVDV-1 and -2 [32,36–38] that might be preferred by particular virus strains showing CD46_bov_-independent entry [34] (addressed in more detail in the Discussion section).

Passaging of CSFV in cell culture can lead to increased usage of the cellular glycosaminoglycan (GAG) heparan sulfate (HS) as an attachment factor [39–41]. In principle, cell culture adaptation mediated by increased HS binding rarely occurs in BVDV. However, especially under selective pressure through lack of CD46_bov_, BVDV-1 and BVDV-2 are also able to adapt to HS [42,43]. Responsible for this adaptation is a mutation in the E^rns^ protein that facilitates enhanced binding to HS [39,43,44], making adapted BVDV-1 and -2 strains independent of their original receptor CD46_bov_ [43].

For both HoBiPeV and GPeV, very little is known about their interaction with the host cell. The aim of this study was to investigate the role of CD46_bov_ in the entry of pestiviruses infecting bovine hosts, namely HoBiPeV and GPeV. Performing loss-of-function and gain-of-function experiments, we were able to show that CD46_bov_ is a major cellular entry factor for HoBiPeV strain HaVi-20, a member of the species *Pestivirus H*. In contrast, CD46_bov_ appears to play only a minor role in the entry of GPeV strain PG-2 representing *Pestivirus G*, indicating that this species may prefer another so far unidentified receptor or compensate the absence of CD46_bov_ by using a different entry mechanism involving other molecular determinants.

## Material and methods

### Cells and viruses

Madin-Darby bovine kidney (MDBK) cells were obtained from the American Type Culture Collection, Manassas, VA, USA (ATCC CCL-22). 293T cells originated from the Leibniz Institute DSMZ – German Collection of Microorganisms and Cell Cultures, Braunschweig, Germany (ACC 635). Cells were maintained in Dulbecco’s Modified Eagle’s Medium (DMEM) supplemented with 1000 U/ml penicillin, 50 µg/ml streptomycin and either 10% horse serum for MDBK (and derivates generated in this study) or 10% fetal calf serum (FCS) for 293T cells at 37°C and 5% CO_2_.

BVDV-1 strain NADL and BVDV-2 strain CS8644 came from the pestivirus collection at the Institute of Virology, University of Veterinary Medicine, Hannover, Germany. HoBiPeV strain D32/00_“HoBi” (hereinafter referred to as HoBi) was a kind gift from Horst Schirrmeier (Institute of Diagnostic Virology, Friedrich-Loeffler-Institute, Greifswald, Germany). A second virus strain belonging to *Pestivirus H* was isolated from contaminated FCS within this study and designated “Hannover-Virology-2020” (HaVi-20). The two virus strains representing the species *Pestivirus G*, H138 and PG-2, have been described previously [27,45,46].

### Generation of an MDBK CD46-knockout cell line

**Figure 1.**
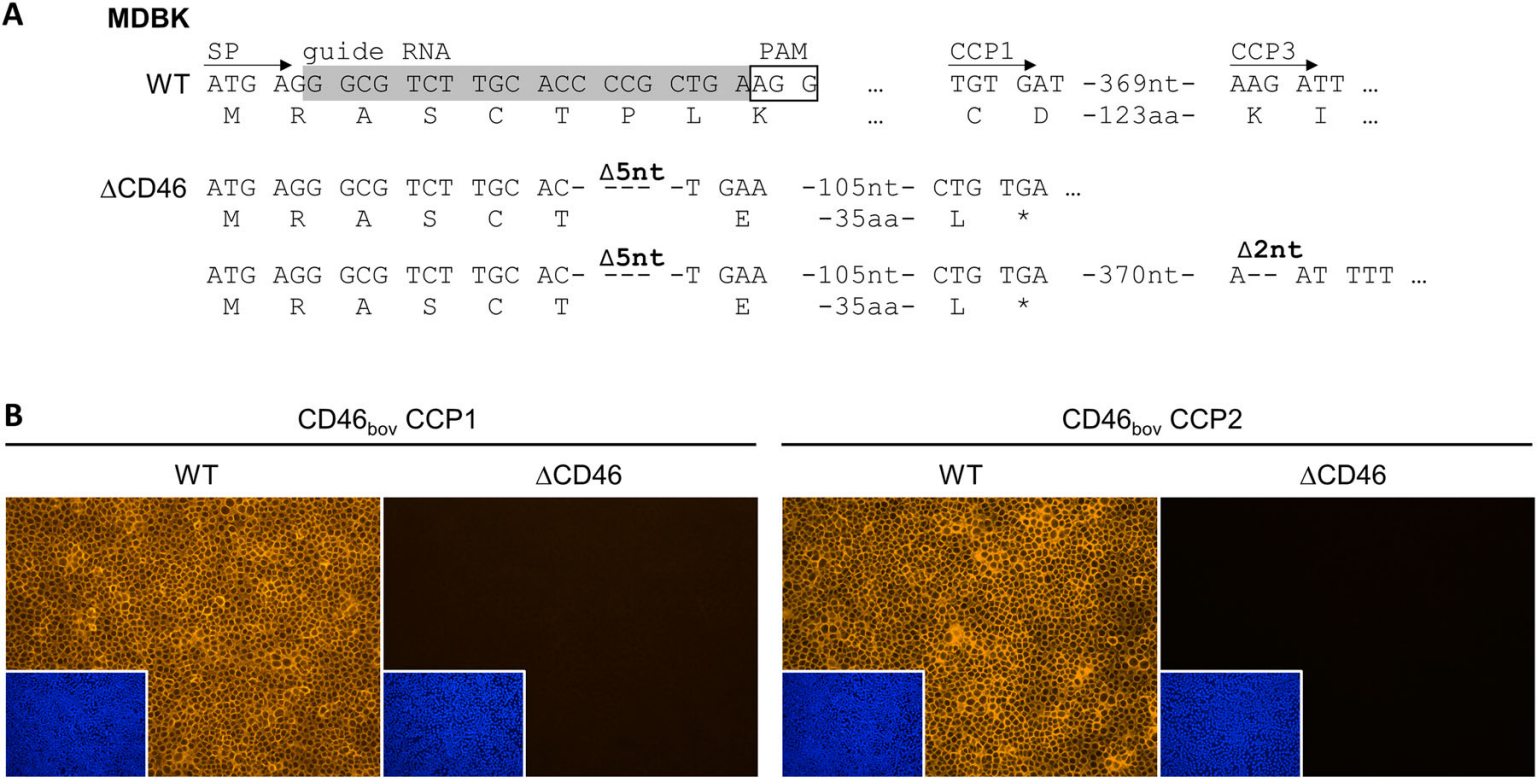
Genome editing strategy and characterization of MDBK CD46-knockout cells. (A) CRISPR/Cas9 genome editing strategy and genetic characterization of generated MDBK CD46-knockout (MDBKΔCD46) cells. The MDBK wild type (WT) consensus nucleotide sequence and deduced amino acid sequence are shown in the top rows. Sequences encoding the CD46_bov_ signal peptide (SP), the complement control protein domains 1 and 3 (CCP1 and -3) as well as the guide RNA (highlighted in grey) and its protospacer adjacent motif (PAM, boxed) are indicated. Nucleotide sequences obtained from MDBKΔCD46 (ΔCD46) cells and resulting amino acid sequences are shown below. Deletions of five and two nucleotides (Δ5nt and Δ2nt, respectively) and stop codons (*) are indicated. (B) Phenotypic characterization of MDBKΔCD46 cells. CD46_bov_ expression of MDBK WT and MDBKΔCD46 cells was detected by immunofluorescence staining of CCP1 (mab CA 26/2/5) and CCP2 (mab CA 17/2/1). Nuclei were stained with DAPI (blue) to visualize the presence of confluent cell monolayers (small pictures in lower left corners).

MDBK CD46-knockout (MDBKΔCD46) cells were generated by CRISPR/Cas9 technology as described before [47,48]. Briefly, two complementary oligonucleotides (gbovCD46_fw and gbovCD46_rev, Table 1) containing the guide sequence targeting the region encoding for the signal peptide (SP) of CD46_bov_ were designed (Figure 1(A)). Primers were phosphorylated, annealed to each other and cloned into the lentiviral transfer vector pLentiCRISPR-v2 [49], resulting in pLentiCRISPR-v2_CD46_bov_.

**Table 1.** Oligonucleotides / primers used in this study.

Name	Sequence (5’→3’)	Target	Application
gbovCD46_fw	CACCG**GGCGTCTTGCACCCCGCTGA**	SP	Generation of CD46-knockout cells
gbovCD46_rev	AAAC**TCAGCGGGGTGCAAGACGCC**C	SP	
bovCD46_14-fw	GTCTCTGCGGAGCAGTCTTG	SP	Genetic characterization of CD46-knockout cells
bovCD46_744-rev	GGGTTGACTACTCCATTCACC	CCP3	
Giraffe-Erns_1034-fw	GTCAAGAAGAAGGGTAAGGTC	Core	Amplification and sequencing of E^rns^
BDV-Erns_1988-rev	CCATCCTCCGCATTGGTGTC	E1	
*Sbf*I_bovCD46-fw	TAAACCTGCAGGATGAGAGCATCCTG CACACCTCTAAAGGCGCCGCTCCG	SP	Generation of CD46-rescue cells
*Mlu*I_bovCD46-rev	TAGTACGCGTTCAGTTCATCTGTTCTG TTGCAGTGGTTGCTTTATCCTGATAAG	CT	
M13-fw	GTAAAACGACGGCCAG	pCR2.1-TOPO plasmid	Sequencing of TOPO plasmids containing CD46_bov_ variants
M13-rev	CAGGAAACAGCTATGAC	pCR2.1-TOPO plasmid	

Bold: CRISPR/Cas9 guide RNA sequence.

Underlined: silent mutations for alteration of guide RNA target sequence.

SP, CCP3, CT: sequences encoding for the signal peptide (SP), complement control protein 3 (CCP3), cytoplasmic tail (CT) of CD46_bov._

Core, E^rns^, E1: sequences encoding for the core, E^rns^, E1 protein of giraffe pestivirus (GPeV).

For the production of lentiviral particles, pLentiCRISPR-v2_CD46_bov_, a packaging plasmid (pCMVΔR8.91) [50], and an envelope plasmid (pMD.G) [51] were co-transfected into 293T cells using polyethylenimine (PEI; 24765-1, Polysciences). Lentiviral particles were harvested 72 h post transfection and used for transduction of MDBK cells. Selection was carried out with 5 µg/ml puromycin (P8833, Sigma-Aldrich) for two weeks.

In order to generate a clonal cell line, single cells were picked and expanded. Absence of CD46_bov_ in the obtained cell cultures was evaluated by immunofluorescence (IF) staining of CD46_bov_ as described below. One CD46-negative cell line was subjected to a second round of biological cloning and characterized as described below.

### Genetic and phenotypic characterization of MDBKΔCD46 cells

In order to characterize the CRISPR/Cas9-induced genome alterations of CD46_bov_ in the MDBKΔCD46 cell line, cellular RNA was extracted from MDBK wild type (WT) and MDBKΔCD46 cells using the KingFisher™ Duo Prime Purification System (Thermo Fisher Scientific) and the IndiMag® Pathogen Kit (SP947457, Indical Bioscience) according to the manufacturers’ instructions. cDNA synthesis was performed with SuperScript™ III Reverse Transcriptase (18080093, Invitrogen) and random hexamer primers (48190011, Invitrogen). To obtain templates for sequencing, a 731 bp amplicon was generated using the ALLin™ HS Red Taq Mastermix (HSM0305, highQu) and a primer pair flanking the guide RNA target site (bovCD46_14-fw and bovCD46_744-rev, Table 1). The following thermal profile was applied: 95°C for 3 min, 40 cycles of 95°C for 15 sec, 56°C for 30 sec and 72°C for 30 sec, and final extension at 72°C for 5 min. PCR amplicons were purified using the GeneJET PCR Purification Kit (K0702, Thermo Fisher Scientific). Purified amplicons from RNA of MDBK WT cells were directly subjected to Sanger sequencing conducted by LGC Genomics (Berlin, Germany) to generate a consensus sequence of the CD46_bov_ encoding mRNA.

Purified amplicons from MDBKΔCD46 cells were cloned into the pCR™ 2.1-TOPO™ TA-vector using the TOPO™ TA Cloning™ Kit (450641, Invitrogen). Plasmids were propagated in *E. coli* TOP10 bacteria. Subsequently, individual colonies were picked and further propagated in over-night cultures. Plasmids were purified using the QIAprep Spin Miniprep Kit (27106, QIAGEN) and subjected to Sanger sequencing. Analysis of 18 obtained sequences was performed with GENtle (version 1.9.4) to determine genetic alterations of CD46_bov_ in comparison to the MDBK WT consensus sequence.

Expression of CD46_bov_ in MDBK WT and MDBKΔCD46 cells was phenotypically characterized by IF staining of CD46_bov_. Cells were grown in 24-well plates for three days, washed once with phosphate-buffered saline (PBS) and fixed at 80°C for 4 h. For staining, two monoclonal antibodies (mabs) specific for epitopes in two different domains of CD46_bov_ were used: CA 26/2/5 (formerly designated BVD/CA 26/2/5), directed against CCP1 (diluted 1:100 in PBS+0.05% Tween), and CA 17/2/1 (formerly designated BVD/CA 17/2/1), directed against CCP2 (diluted 1:50) [32,34]. As secondary mab, Cy3-AffiniPure goat anti-mouse IgG (115-165-146, Dianova; diluted 1:800) was used and cell nuclei were stained with 4’,6-diamidino-2-phenylindole (DAPI; D3571, Thermo Fisher Scientific; diluted 1:500). IF analysis was carried out using a Leica DMI3000 B microscope (Leica Microsystems). Pictures were taken with a Leica DFC3000 G camera using the Leica Application Suite software (version 4.13.0).

### Infection experiments

The role of CD46_bov_ as a cellular receptor for different bovine pestiviruses was determined by *in vitro* infection experiments. The respective cells were seeded in 24-well plates one day prior to infection. On the day of infection, cells were trypsinized and counted. In order to achieve adequate and comparable infection levels of WT cells at 16 h post infection (hpi), the multiplicity of infection (MOI) was adjusted to 1 for all virus strains, with the exception of HoBiPeV strain HaVi-20 (MOI = 10) and GPeV strain PG-2 (MOI = 2). Cells were incubated with the virus dilutions at 37°C for 2 h. Subsequently, inocula were removed and cells were washed three times with PBS before adding 1 ml fresh media and further incubation at 37°C. At 16 hpi, supernatants were harvested and frozen at −80°C until further analysis. Cells were washed once with PBS and heat-fixed at 80°C for 4 h. All infection experiments were repeated three times.

### Immunofluorescence assay

Viral infections were visualized by IF staining using mab C16, which was raised against the BVDV NS3 protein and is broadly reactive with most pestiviruses [52,53]. Heat-fixed cells were stained with mab C16 diluted 1:50 in PBS+0.05% Tween-20 at 37°C for 1 h, followed by secondary mab Cy3-AffiniPure goat anti-mouse IgG (115-165-146, Dianova) diluted 1:800 at 37°C for 1 h. Cell nuclei were stained with 4’,6-diamidino-2-phenylindole (DAPI; D3571, Thermo Fisher Scientific; diluted 1:500).

For quantification of positive signals in IF, five pictures per well, in total representing approx. 15% of the infected monolayer in one well, were taken. Using the ImageJ software (version 1.51q), images were converted into binary, and positive pixels were counted applying the same threshold for all pictures that were compared with each other.

### Heparan sulfate adaptation assay

In order to avoid misinterpretation regarding the relevance of CD46_bov_ as a receptor for the investigated pestiviruses, their cell culture adaptation to heparan sulfate (HS) was investigated first. Therefore, virus dilutions were pre-incubated with 2000 µg/ml heparin (H3149-10KU, Sigma-Aldrich) at 37°C for 30 min prior to infection to block HS binding sites located on the viral envelope proteins as described previously [39]. Infection was conducted as described above.

### E^rns^ sequence analysis

GPeV strain PG-2 RNA was prepared from the virus stock used for infection experiments using the QIAamp Viral RNA Mini Kit (52904, QIAGEN). cDNA synthesis and generation of a 959 bp amplicon covering the E^rns^ gene were performed as described above for the genetic characterization of MDBKΔCD46 cells using primers Giraffe-Erns_1034-fw and BDV-Erns_1988-rev (Table 1). Purification of PCR amplicons, Sanger sequencing and sequence analysis were conducted as described above.

### CD46_bov_ blocking assay

In order to confirm the results obtained with the MDBKΔCD46 cells with a second method, a CD46_bov_ blocking assay was performed using mab CA 17/2/1 (isotype IgG2a) directed against the CCP2 domain of CD46_bov_ which has been applied for blocking BVDV-1 infection previously [32,38]. Prior to infection, cells were washed once with PBS and incubated with hybridoma supernatant containing mab CA 17/2/1 (diluted 1:2) or a non-relevant hybridoma supernatant (BM 40/1/10; diluted 1:2) as control at 37°C for 1 h. Subsequently, cells were washed twice and infection was performed as described above. After 2 h, cells were washed twice before adding fresh media containing the same amount of the respective mab as used for blocking prior to infection. For IF staining of pestiviruses, mab C16 (isotype IgG1) was used in combination with isotype-specific secondary mab goat IgG anti-mouse IgG1 (Fc)-Cy3 (115-165-205, Dianova; diluted 1:800) not detecting the isotype IgG2a mab CA 17/2/1 used for blocking of CD46_bov_.

### Generation and characterization of stable MDBKΔCD46 rescue cell lines expressing CD46_bov_

MDBKΔCD46 cell lines were trans-complemented using recombinant lentiviruses for stable expression of two different CD46_bov_ transcript variants in order to exclude off-target effects and confirm the role of CD46_bov_ for infections with the investigated pestiviruses. Methodological details about the lentiviral transduction system have been described elsewhere [47]. Briefly, cellular RNA was isolated and reverse-transcribed as described above. The complete CD46_bov_ mRNA sequence was amplified using the ALLin™ HS Red Taq Mastermix, applying the following thermal profile: 95°C for 3 min, 28 cycles of 95°C for 15 sec, 52.5°C for 30 sec and 72°C for 30 sec, and final extension at 72°C for 5 min. Primers *Sbf*I_bovCD46-fw and *Mlu*I_bovCD46-rev contained synonymous mismatches to the wild type sequence to modify the sequence targeted by the CRISPR/Cas9 enzyme (Table 1, Figure 4(A)). At the same time, *Sbf*I and *Mlu*I restriction sites were added to the ends of the amplicons, respectively. Purified PCR amplicons were cloned into the TOPO vector and propagated in *E. coli* TOP10 bacteria. Multiple colonies were propagated and plasmids were purified and subjected to Sanger sequencing with vector-specific primers M13-fw and M13-rev (Table 1). Sequence analysis revealed different CD46_bov_ transcript variants present in the MDBK WT cells, of which two were selected for further use: one with a long cytoplasmic tail (CD46-CTL) and one with a short cytoplasmic tail (CD46-CTS). They also differed in the composition of the ectodomain and length of the STP domain (Figure 4(B)). The CD46-CTL and CD46-CTS sequences were cloned into the lentiviral plasmid vector pWPI-msc-GUN [54] via *Sbf*I and *Mlu*I restriction sites using the T4 DNA Ligase (EL0011, Thermo Scientific). The resulting plasmids pWPI-GUN_CD46-CTL and pWPI-GUN_CD46-CTS were propagated in NEB® Stable Competent *E. coli* (C3040I, New England Biolabs). Sanger sequencing confirmed correct sequences.

For lentiviral transduction, 293T cells were co-transfected with pCMVΔR8.91, pMD.G and either pWPI-GUN_CD46-CTL or pWPI-GUN_CD46-CTS, respectively. Lentiviral particles were used to transduce MDBKΔCD46 cells, followed by selection with 1000 µg/ml Geneticin (10131035, Gibco) for two weeks.

To confirm the expression of CD46-CTL and CD46-CTS, respectively, both MDBKΔCD46 rescue cell lines were characterized phenotypically by IF staining of CD46_bov_ as described above for MDBK WT and MDBKΔCD46 cells.

### Virus titration

Viral titres in supernatants from infection experiments were determined by endpoint dilution assay with three-fold virus dilutions incubated on MDBK cells. After 72 h, cytopathic effect (CPE) was assessed and cells were heat-fixed as described above. Titrations were evaluated by IF staining using mab C16 in combination with mab Cy3-AffiniPure goat anti-mouse IgG as described above. The 50% tissue culture infective dose per ml (TCID_50_/ml) was calculated using the Spearman-Karber method [55,56]. All titrations were performed in quadruplicate and repeated three times.

### Statistical analysis

Data were analysed with Microsoft Excel, R (version 4.1.0, https://www.r-project.org) and SAS (version 9.4). For comparison of two groups, nonparametric permutation tests [57] and for comparison of multiple groups, one-way ANOVAs with multiple comparison procedures (Tukey) were applied for original values. Based on the calculated p-values, observed differences were interpreted as significant (*p < 0.05), very significant (**p < 0.01) or extremely significant (***p < 0.001).

## Results

### Generation and characterization of the MDBK CD46-knockout cell line

MDBK cells deficient for CD46_bov_ (MDBKΔCD46) were generated to investigate the role of CD46_bov_ as a cellular receptor for different bovine pestiviruses (Figure 1).

In order to characterize the genomic modifications generated by CRISPR/Cas9-based genome editing, PCR amplicons from the N-terminal region of CD46_bov_ were sequenced. Analysis of sequences from 18 independent cDNA clones revealed the presence of two different variants confirming genome alterations on two alleles. A small deletion of five nucleotides that leads to a frameshift in the sixth codon of the signal peptide (SP) was identified on both alleles, resulting in only five unchanged amino acids (aa) left at the N-terminus of the protein. One allele has an additional deletion of two nucleotides within the sequence encoding the CCP3 domain, also resulting in a frameshift but not restoring the original reading frame (Figure 1(A)).

For phenotypic characterization of the MDBKΔCD46 cells by IF staining of CD46_bov_, two different antibodies were used: one directed against the CCP1 domain and the other one directed against the CCP2 domain of CD46_bov_. With neither antibody could CD46_bov_ be detected, indicating that, in contrast to the MDBK WT cells, the MDBKΔCD46 cell line does not express CD46_bov_ (Figure 1(B)).

### Heparan sulfate adaptation of *Pestivirus H* and *Pestivirus G* strains

**Figure 2.**
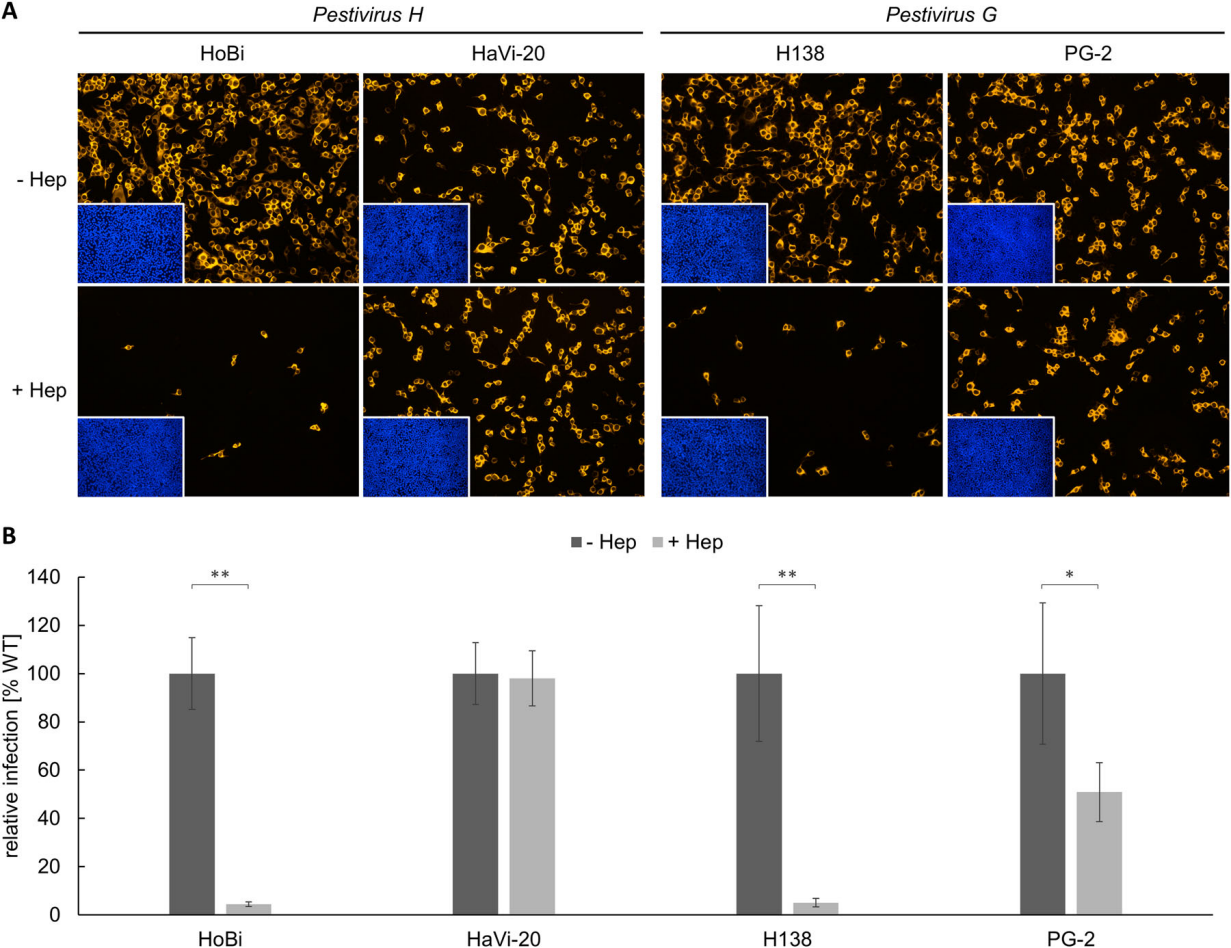
Heparan sulfate adaptation of *Pestivirus H* and *Pestivirus G* strains used in this study. (A) MDBK wild type (WT) cells were infected with HoBi-like pestivirus strains HoBi and HaVi-20, and giraffe pestivirus strains H138 and PG-2. Native infections (- Hep, top row) were compared with infections after pre-incubation of virus inocula with heparin (+ Hep, bottom row), respectively. For immunofluorescence analysis, pestivirus non-structural protein NS3 was visualized using mab C16 in combination with secondary mab Cy3-AffiniPure goat anti-mouse IgG (orange). Nuclei were stained with DAPI (blue) to visualize the presence of confluent cell monolayers (small pictures in lower left corners). (B) Infections were quantified by pixel counting using the ImageJ software. Bars represent mean values from five pictures per well. Standard deviations are indicated. Native infections (- Hep) were set as 100% and infections after pre-incubation with heparin (+ Hep) were put in relation. Significance was calculated using a permutation test (*p < 0.05; **p < 0.01).

As cell culture adaptation to HS might obscure the relevance of CD46_bov_ for viral entry, HS usage of *Pestivirus H* and *Pestivirus G* strains used in this study was investigated first. Infection of MDBK WT cells was evaluated and quantified at 16 hpi by IF analysis (Figure 2).

After blocking HS binding sites on the viral envelope proteins with heparin, infection with HoBiPeV strain HoBi was reduced by approx. 95%, indicating a strong cell culture adaptation and a high degree of HS dependency. In order to obtain a non-cell-culture-adapted *Pestivirus H* strain, a new virus strain was isolated directly from contaminated commercial FCS. In contrast to strain HoBi, the third passage of the new virus isolate HaVi-20 was hardly affected by heparin showing only a slight reduction of approx. 2%.

Comparable to HoBiPeV strain HoBi, a high level (approx. 95%) of cell culture adaptation and HS usage was also observed for GPeV strain H138. Interestingly, infection with strain PG-2 suggested a lower degree of cell culture adaptation. About half of the virus population was able to enter the cells independently of HS, probably using its original receptor(s) (Figure 2). Sequencing of the region encoding E^rns^ of strain PG-2 revealed a single nucleotide polymorphism (SNP) within the codon for aa residue 475 (reference sequence: GenBank accession no. AHW57610.1) resulting in either glycine (uncharged) or arginine (charged), indicating a mixed population of WT and adapted viruses (Figure S1).

### Impact of CD46-deficiency on entry of *Pestivirus H* and *Pestivirus G* strains

**Figure 3.**
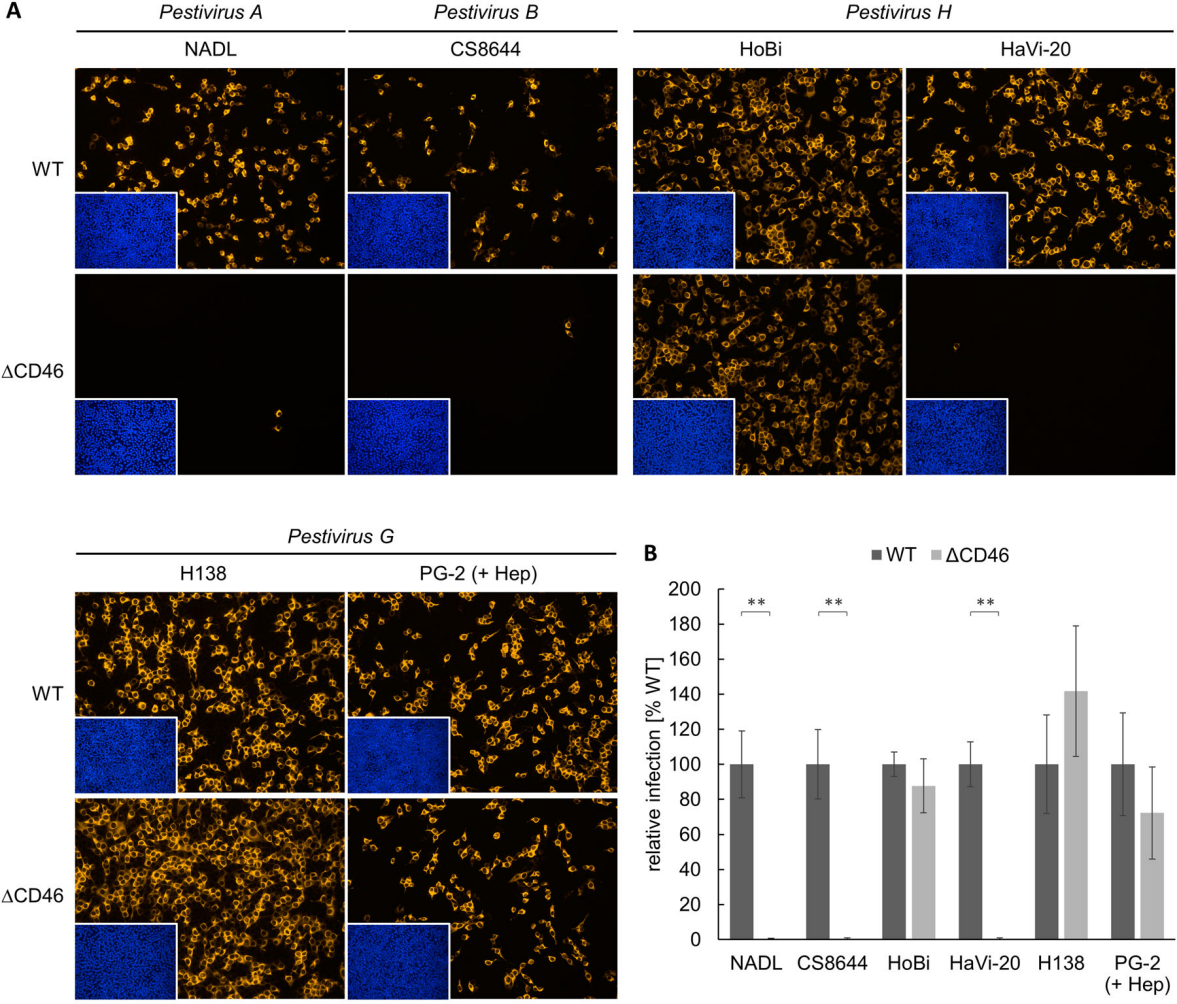
Impact of CD46-deficiency on entry of *Pestivirus H* and *Pestivirus G* strains. (A) MDBK wild type (WT) and MDBK CD46-knockout (ΔCD46) cells were infected with BVDV-1 strain NADL, BVDV-2 strain CS8644, HoBi-like pestivirus strains HoBi and HaVi-20, and giraffe pestivirus strains H138 and PG-2 for 16 h. Partially cell-culture-adapted PG-2 was pre-incubated with heparin (+ Hep) to block binding to heparan sulfate. Immunofluorescence staining of pestivirus non-structural protein NS3 was performed using mab C16 in combination with secondary mab Cy3-AffiniPure goat anti-mouse IgG (orange). Nuclei were stained with DAPI (blue) to visualize the presence of confluent cell monolayers (small pictures in lower left corners). (B) Infections were quantified by pixel counting using the ImageJ software. Bars represent mean values from five pictures per well. Standard deviations are indicated. Infections of MDBK WT cells were set as 100% and infections of MDBKΔCD46 cells were put in relation. Significance was calculated using a permutation test (**p < 0.01).

In order to characterize the role of CD46_bov_ in the entry of bovine pestiviruses, MDBK WT and generated MDBKΔCD46 cells were infected with BVDV-1 strain NADL, BVDV-2 strain CS8644, HoBiPeV strains HoBi and HaVi-20, and GPeV strains H138 and PG-2, respectively. Infection was evaluated and quantified at 16 hpi by IF analysis (Figure 3).

As expected, BVDV-1 strain NADL and BVDV-2 strain CS8644 infection of MDBKΔCD46 cells were dramatically reduced in comparison to MDBK WT cells, with only very few infected cells left (reduction > 99%). Infection of MDBKΔCD46 cells with HoBiPeV strain HaVi-20 was reduced to a similar extent as the BVDV-1 and BVDV-2 strains used as controls, indicating that CD46_bov_ is an essential cellular entry factor also for viruses belonging to *Pestivirus H*. As GPeV strain PG-2 was shown to be partially cell-culture-adapted, it was pre-incubated with heparin to block binding to HS. Interestingly, infection of MDBKΔCD46 cells was reduced by only approx. 28% in comparison to MDBK WT cells, suggesting that CD46_bov_ plays a rather minor role in the entry of viruses representing *Pestivirus G*. HoBiPeV strain HoBi and GPeV strain H138 were shown to be cell-culture-adapted. Consequently, their infectivity was not affected by CD46-deficiency (Figure 3).

The different role of CD46_bov_ as a receptor for *Pestivirus H* and *Pestivirus G* strains as determined by CD46-knockout was confirmed by antibody-mediated blocking of CD46_bov_ on the surface of MDBK cells (Figure S2). Blocking of CD46_bov_ almost completely inhibited infection with BVDV-1 strain NADL, BVDV-2 strain CS8644 as well as HoBiPeV strain HaVi-20. In contrast, susceptibility to GPeV strain PG-2 was hardly affected by antibody-mediated blocking (Figure S2).

### Generation and characterization of stable MDBKΔCD46 rescue cell lines expressing CD46_bov_

**Figure 4.**
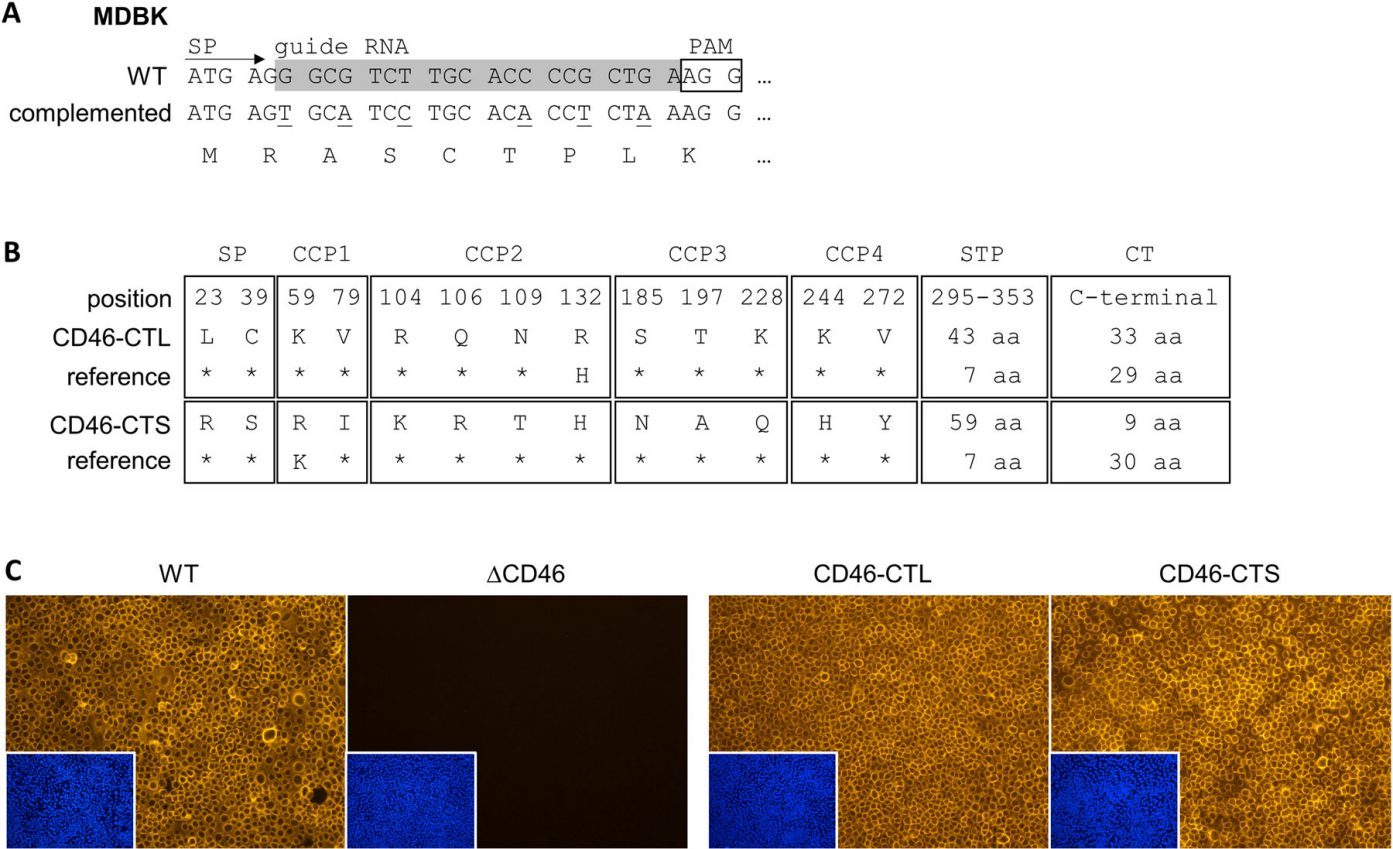
Trans-complementation strategy and characterization of MDBKΔCD46 rescue cell lines expressing CD46_bov_. (A) For trans-complementation of CD46_bov_ via lentiviral transduction, the guide RNA target sequence within the signal peptide (SP) coding region was altered by introduction of six silent mutations (underlined nucleotides in “complemented” sequence). (B) Different domains of CD46_bov_ including signal peptide (SP), complement control protein 1–4 (CCP1-4), Serine/Threonine/Proline-rich region (STP) and cytoplasmic tail (CT) are shown. Trans-complementation was performed using recombinant lentiviruses carrying two genetically distinct CD46_bov_ transcript variants, one with a long (CD46-CTL: 33 aa) and one with a short (CD46-CTS: 9 aa) CT. Apart from their CTs, CD46-CTL and -CTS differ in 13 aa within SP-CCP4 as well as in the length of the variable STP domain. Most similar SP-CCP4 sequences identified in GenBank (reference; CD46-CTL: DAA20944, CD46-CTS: AIC33504) differ in only one aa. Residues identical to CD46-CTL and CD46-CTS are indicated, respectively (*). (C) Phenotypic characterization of MDBKΔCD46 CD46-rescue cell lines. CD46_bov_ expression of MDBK WT (WT), MDBKΔCD46 (ΔCD46) and CD46-rescue cell lines (CD46-CTL, CD46-CTS) was detected by immunofluorescence staining of CCP1 (mab CA 26/2/5). Nuclei were stained with DAPI (blue) to visualize the presence of confluent cell monolayers (small pictures in lower left corners).

In order to exclude off-target effects and to demonstrate gain-of-function, non-permissive CD46-knockout cells were trans-complemented with CD46_bov_ (Figure 4(A)). Considering the diverse amino acid sequences and different splice variants of CD46_bov_, two cell lines expressing very distinct variants present in the highly permissive MDBK WT cells were established. The selected CD46_bov_ variants contained several distinct amino acids in their ectodomains but also differed in length and composition of their STP domains and CTs. One CD46_bov_ variant comprised a long (CD46-CTL) and one a short cytoplasmic tail (CD46-CTS) (Figure 4(B)), as it was reported that the length of the CT of CD46_bov_ can shift permissivity to BVDV-1 strain NADL [35].

Phenotypic characterization of the generated MDBKΔCD46 + CD46-CTL and MDBKΔCD46 + CD46-CTS cell lines by IF staining revealed that both cell lines express CD46_bov_ at least as strongly as the WT cells (Figure 4(C)).

### Impact of CD46-rescue on entry of *Pestivirus H* and *Pestivirus G* strains

**Figure 5.**
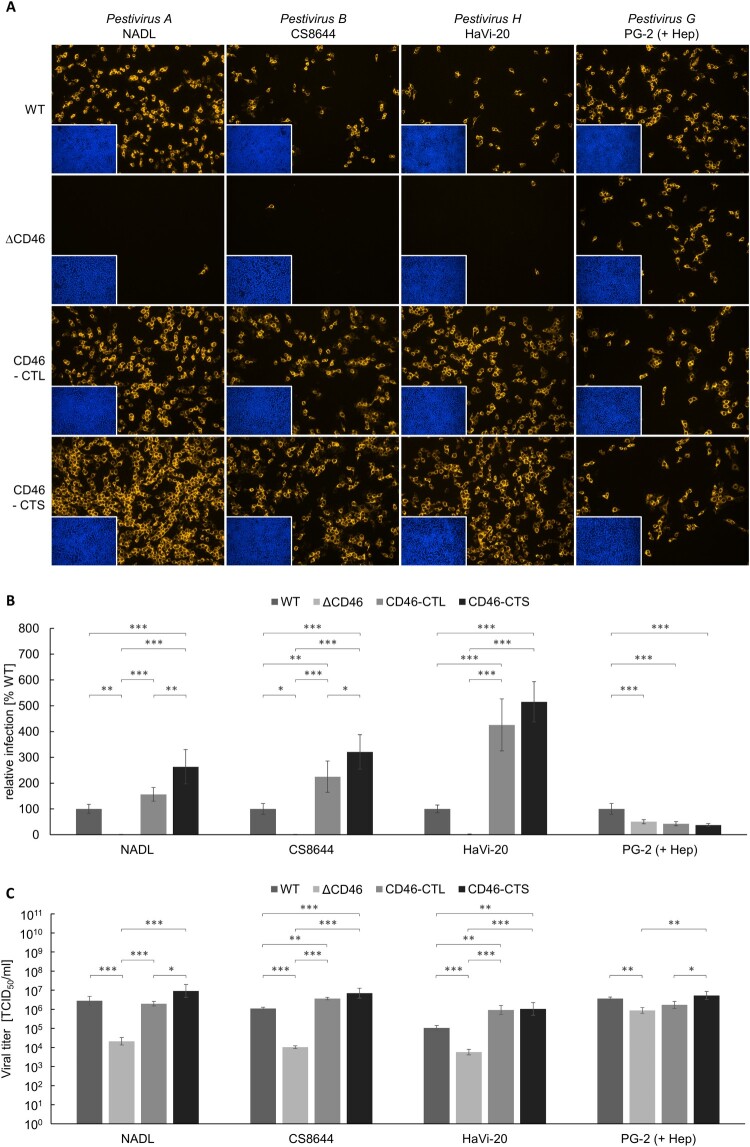
Impact of CD46-rescue on entry of *Pestivirus H* and *Pestivirus G* strains. (A) MDBK wild type (WT), MDBK CD46-knockout (ΔCD46) and MDBKΔCD46 cells trans-complemented with CD46_bov_ comprising either a long (CD46-CTL) or a short cytoplasmic tail (CD46-CTS) were infected with BVDV-1 strain NADL, BVDV-2 strain CS8644, HoBi-like pestivirus strain HaVi-20 and giraffe pestivirus strain PG-2 and incubated for 16 h, respectively. Partially cell-culture-adapted strain PG-2 was pre-incubated with heparin (+ Hep) to block binding to heparan sulfate. Immunofluorescence staining of pestivirus non-structural protein NS3 was performed using mab C16 in combination with secondary mab Cy3-AffiniPure goat anti-mouse IgG (orange). Nuclei were stained with DAPI (blue) to visualize the presence of confluent cell monolayers (small pictures in lower left corners). (B) Infections were quantified by pixel counting using the ImageJ software. Bars represent mean values from five pictures per well. Standard deviations are indicated. Infections of MDBK WT cells were set as 100% and infections of the other cell lines were put in relation. (C) Infectious viral titres were determined by titration of supernatants from infection experiments. Bars represent mean 50% tissue culture infectious doses per ml (TCID_50_/ml) from three titrations each performed in quadruplicate. Standard deviations are indicated. (B+C) Significance was tested using one-way ANOVAs with Tukey post-hoc tests (*p < 0.05; **p < 0.01; ***p < 0.001).

Virus strains that showed a dependency on CD46_bov_ when comparing infection of MDBK WT and MDBKΔCD46 cells were further studied by infecting MDBKΔCD46 cells rescued with CD46-CTL or CD46-CTS. Infection and viral replication were analysed at 16 hpi by IF analysis and titration of cell culture supernatants, respectively (Figure 5).

Permissivity to BVDV-1 strain NADL, BVDV-2 strain CS8644 and HoBiPeV strain HaVi-20 was significantly diminished by CD46-knockout reflected by an approx. 20-fold (HaVi-20) to 100-fold (NADL and CS8644) decrease in viral titres compared to titres produced in MDBK WT cells. Trans-complementation of MDBKΔCD46 cells with either of the CD46_bov_ variants restored permissivity. In comparison to MDBK WT cells, trans-complementation with CD46-CTL resulted in an equally strong or slightly enhanced permissivity producing the same (NADL) or approx. three-fold (CS8644; **p < 0.01) to 10-fold (HaVi-20; **p < 0.01) increased viral titres. For HoBiPeV strain HaVi-20, expression of CD46-CTL and CD46-CTS resulted in equal permissivity. Interestingly, for BVDV-1 strain NADL and BVDV-2 strain CS8644, trans-complementation with CD46-CTS enabled a more efficient infection than CD46-CTL, with approx. three-fold (NADL) and six-fold (CS8644; ***p < 0.001) increased titres in comparison to MDBK WT cells.

In accordance with the low impact CD46-knockout and antibody-mediated blocking of CD46_bov_ had on GPeV strain PG-2 infectivity, CD46-rescue also had only a weak effect on the permissivity of the cells to strain PG-2. Its titre was only slightly (approx. four-fold) decreased on MDBKΔCD46 cells in comparison to strong (20-fold to 100-fold) decreases for the other investigated viruses. Trans-complementation with CD46-CTL or CD46-CTS had no clear effect on permissivity to GPeV strain PG-2 (Figure 5).

## Discussion

The host cell entry is a crucial step in the viral replication cycle; however, the pestiviral entry process is still poorly understood. BVDV-1 (*Pestivirus A*) and BVDV-2 (*Pestivirus B*) use CD46_bov_ as a cellular receptor [32]. Based on antibody-mediated blocking, it was suggested that CSFV (*Pestivirus C*) uses porcine CD46 (CD46_pig_) as a major cellular entry factor [40]. A recent study using porcine CD46-knockout cells revealed that CSFV as well as Bungowannah pestivirus (BuPV; *Pestivirus F*) can efficiently enter porcine host cells independently of CD46_pig_, while it is an essential entry factor for Atypical porcine pestivirus (APPV; *Pestivirus K*) [48]. These results demonstrate that the entry mechanisms of pestiviruses are more diverse than previously assumed. In the present study, we investigated the role of CD46_bov_ as a cellular receptor for virus strains of additional pestivirus species that can efficiently infect bovine host cells, namely *Pestivirus H* and *Pestivirus G*.

The role of CD46_bov_ as a receptor for BVDV-1 and -2 was demonstrated by blocking infections using anti-CD46_bov_ mabs or a polyclonal rabbit anti-CD46_bov_ serum and by CD46-transfection of non-permissive porcine cells [32,34]. Meanwhile, CRISPR/Cas9 technology is widely used. It enables the generation of knockout cells and provides an elegant tool for studying cellular factors involved in viral entry. In a recent study, MDBK CD46-knockout cells were used to investigate the ability of BVDV-1 and -2 to escape CD46-dependency [43].

For the generation of MDBKΔCD46 cells, we positioned the CRISPR/Cas9 guide RNA very close to the 5′ end of the CD46_bov_ encoding sequence within the sequence encoding the SP. Genetic and phenotypic characterization confirmed the successful generation of a CD46-negative cell line. CD46-deficiency strongly impaired infections with selected BVDV-1 and -2 strains, which is consistent with the observations of Szillat *et al.* [43]. Within the present study, a similar dependence on CD46_bov_ was shown for isolate HaVi-20, a member of the species *Pestivirus H*. All three viruses exhibited a low residual infectivity that, for BVDV-1 and -2, was also reported after blocking CD46_bov_ with mabs, suggesting the use of at least one additional entry factor [34]. Previously, it was already noted that the ubiquitous expression of CD46_bov_ is not consistent with the tissue tropism of BVDV [32]. There has been further evidence for other molecular determinants involved in entry of BVDV for a long time [36]. The ability of BVDV-1 to infect polarized airway epithelial cells from the basolateral side where no CD46_bov_ is expressed also points to the existence of an alternative entry factor [38]. Future studies will show whether this unknown factor is also the molecular determinant preferentially used by GPeV which in parts turned out to be both CD46_bov_- and HS-independent. As heparin was used in saturating amounts and almost completely blocked entry of the closely related strain H138, it seems unlikely that entry of PG-2 is based on unspecific binding to HS, providing strong evidence for the presence of another specific receptor in addition to CD46_bov_. For CSFV, there are reports on various membrane proteins involved in entry, such as the laminin receptor, the low density lipoprotein receptor, vinculin, integrin β-3 and ADAM-17 [58–60], but it is unclear how these factors interact with CSFV and whether they have a relevance in entry of BVDV. Investigating the entry mechanism of non-HS-adapted pestiviruses that show a low dependency on CD46_bov_ (e.g. GPeV strain PG-2) might be useful for the identification of alternative entry factors, co-receptors or other additional players in the pestivirus-host cell interaction.

Due to possible unspecific binding of the guide RNA, CRISPR/Cas9 technology carries a risk of off-target effects. Therefore, the impact of CD46_bov_ for entry of the investigated viruses was demonstrated not only using MDBKΔCD46 cells, but also by antibody-mediated blocking of CD46_bov_ and was additionally confirmed with MDBKΔCD46 cells trans-complemented with CD46_bov_. The strong (BVDV-1, BVDV-2 and HoBiPeV) or weak (GPeV) dependence on CD46_bov_ observed in the loss-of-function experiments corresponded well with the respective gain-of-function of two different CD46-rescue cell lines. Interestingly, trans-complementation with CD46-CTS comprising a short cytoplasmic tail did not only restore WT-susceptibility, but even enhanced infection with BVDV-1, BVDV-2 and HoBiPeV. In contrast, Zezafoun *et al.* reported that CD46_bov_ transcript variants with a long CT enhance susceptibility to BVDV-1 [35]. The constructs used in the present study were based on native sequences obtained from MDBK WT cells, which did not only differ in their CTs, but also in 13 aa in CCP1-4 as well as in the length of their STP-regions. The high variability of CD46_bov_ makes it difficult to determine the impact of different (transcript) variants with regard to a cell’s permissivity. In the present study, the length of the CT was apparently not the only factor affecting susceptibility to CD46_bov_-dependent bovine pestiviruses. To the best of our knowledge, the expression pattern of different CD46_bov_ variants and their physiological roles in different cell types has not been investigated so far. It is conceivable that these CD46_bov_ variants may have an influence on the efficiency of the entry of CD46_bov_-dependent bovine pestiviruses and thus determine their cell and tissue tropism. Future studies should address the occurrence and frequency of different CD46_bov_ variants in diverse cell types and could thus also provide information about entry of bovine pestiviruses into cell lines other than MDBK, e.g. from the respiratory or reproductive system, which could also be independent of CD46_bov_.

CSFV can adapt to cell culture conditions very rapidly. Although adaptation of BVDV to cell culture conditions occurs rarely, BVDV-1 and BVDV-2 are also able to overcome lack of the CD46_bov_ receptor after only a few passages by using the ubiquitous GAG HS as an attachment factor [39,40,42,43]. Other viruses use HS or heparan sulfate proteoglycans (HSPG) under *in vivo* conditions to enter the host cell, and for some viruses, the biological relevance of binding to HS or HSPGs is still controversial (reviewed in [61]). So far, there is no knowledge about binding of HoBiPeV and GPeV to HS or HSPGs nor its biological relevance, respectively. Blocking of putative HS binding sites on the viral envelope proteins revealed HoBiPeV strain HoBi to be strongly cell-culture-adapted, while strain HaVi-20 directly isolated from fetal calf serum was not. GPeV strain H138 was also strongly adapted, while strain PG-2 showed only a low degree of HS dependency. Most HS-adapted BVDV-1 and -2 strains exhibit a mutation in the E^rns^ gene, leading to an exchange of the uncharged aa at position 479 with a charged residue [43].

The results from the HS adaptation assay and sequencing of the E^rns^ gene of GPeV strain PG-2 indicated that the used virus stock contained a mixed population of WT and cell-culture-adapted viruses. Analysis of the E^rns^ encoding sequence revealed that aa residue 475 in the polyprotein of GPeV strain PG-2 corresponding to aa residue 479 in the polyprotein of BVDV-1 and -2 is likely crucial for cell culture adaptation of GPeV. To confirm the relevance of this aa residue for HS adaptation, investigation of two separate virus populations each carrying one of the observed sequence variants or establishment of a reverse genetic system for PG-2 would be desirable.

In summary, HoBiPeV strain HaVi-20, a member of the species *Pestivirus H*, uses CD46_bov_ as a major cellular entry factor. Interestingly, GPeV strain PG-2 representing *Pestivirus G* was demonstrated to be less dependent on CD46_bov_, indicating a higher preference for an alternative receptor to enter bovine cells. It remains to be investigated whether the receptor binding sites within the E2 protein or whether other viral determinants might be responsible for the observed differences in cell entry. In conclusion, our results demonstrate that there are basic differences in the biology of the members of the growing *Pestivirus* genus. Future studies will focus on the differences in interaction of pestiviruses with the host cell and enhance our knowledge concerning the various entry mechanisms of pestiviruses.

## Supplementary Material

Supplemental Material
