# Supplementary material for: Different impact of bovine complement regulatory protein 46 (CD46bov) as a cellular receptor for members of the species Pestivirus H and Pestivirus G
Source: Emerg Microbes Infect. 2022 Jan 4;11(1):60–72. doi: 10.1080/22221751.2021.2011620 (PMC8741246; doi:10.1080/22221751.2021.2011620)

Supplementary Figure S1. Genetic characterization of *Pestivirus G* strain PG-2 by analysis of the E^rns^ encoding sequence.

(**A**) Scheme of the E^rns^ encoding sequence of bovine viral diarrhea virus 1 (BVDV-1) strain NADL and giraffe pestivirus (GPeV) strain PG-2 including nucleotide (nt) and amino acid (aa) positions in the genome and the polyprotein, respectively (top). The position of the genomic region putatively crucial for cell culture adaptation to heparan sulfate is indicated. Below, the NADL wild type (WT) nt (GenBank NC001461.1) and aa (GenBank NP040937.1) sequence in this E^rns^ region as well as the heparan sulfate-adapted (HS-adapted) variant described by Szillat *et al.* (2020) are shown. Given are all possible codons encoding the aa Arginine which is presumably responsible for HS adaptation. For PG-2, the WT nt (GenBank KJ660072.1) and aa (GenBank AHW57610.1) sequence are shown likewise. The “HS-adapted” nt sequence containing a single nucleotide polymorphism (SNP) and deduced aa sequence identified in the used virus stock are shown in the bottom row. Variant aa residues are displayed in bold. (**B**) Chromatogram of the nt sequence showing the SNP encoding for the G475R mutation in the E^rns^ of partially HS-adapted GPeV strain PG-2.

Supplementary Figure S1


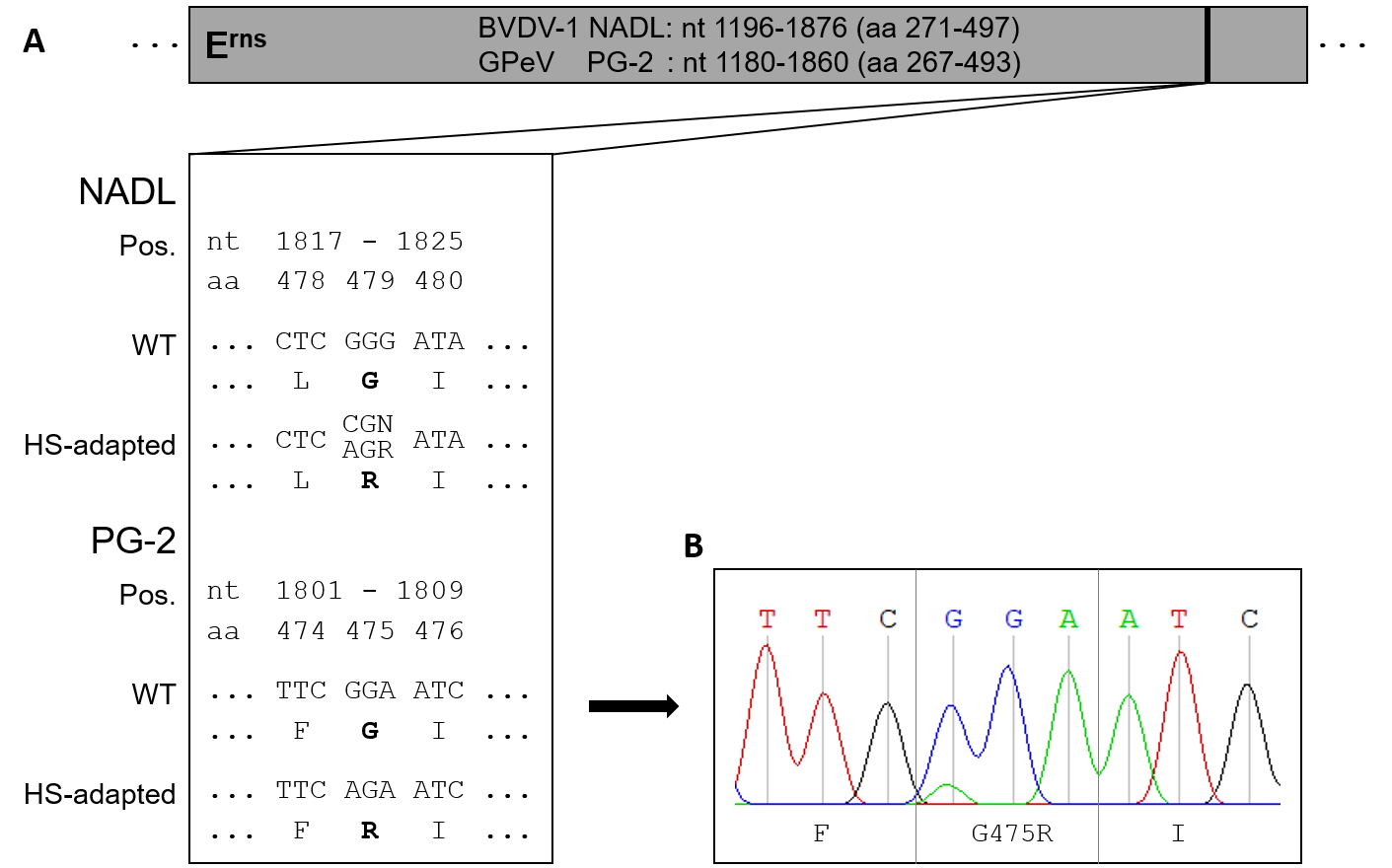


Supplementary Figure S2. Impact of CD46-blocking on entry of *Pestivirus H* and *Pestivirus G* strains.

MDBK wild type (WT) cells were pre-incubated with either non-relevant mab BM 40/1/10 (control, top row) or CD46_bov_-specific mab CA 17/2/1 (anti-CD46_bov_, bottom row) and infected with BVDV-1 strain NADL, BVDV-2 strain CS8644, HoBi-like pestivirus strain HaVi-20 or giraffe pestivirus strain PG-2. Partially cell-culture-adapted strain PG-2 was pre-incubated with heparin (+ Hep) to block binding to heparan sulfate. After infection, cells were incubated for 16 h in presence of the respective mab. Immunofluorescence (IF) staining of pestivirus non-structural protein NS3 was performed using mab C16 in combination with isotype-specific secondary mab goat IgG anti-mouse IgG1 (Fc)-Cy3 (orange). Nuclei were stained with DAPI (blue) to visualize the presence of confluent cell monolayers (small pictures in lower left corners).

Supplementary Figure S2


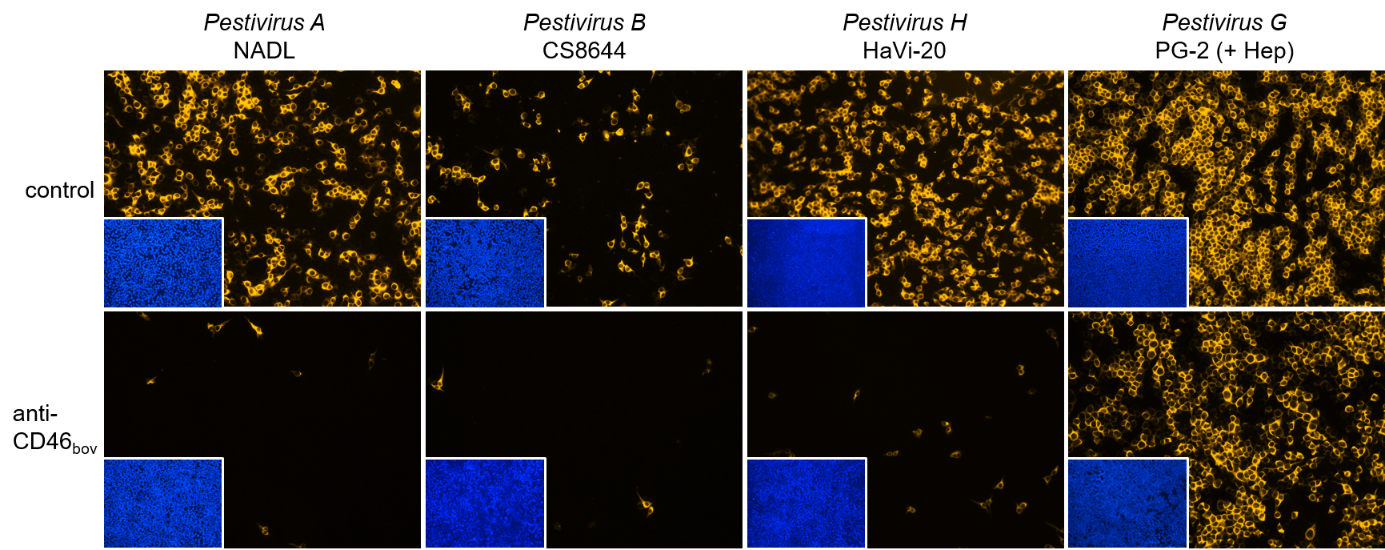

Supplement: Supplemental Material [file TEMI_A_2011620_SM3989.docx]
